# Strong spin-filtering and spin-valve effects in a molecular V–C_60_–V contact

**DOI:** 10.3762/bjnano.3.69

**Published:** 2012-08-22

**Authors:** Mohammad Koleini, Mads Brandbyge

**Affiliations:** 1 Hybrid Materials Interfaces Group, Faculty of Production Engineering and Bremen Center for Computational Materials Science, University of Bremen, 28359 Bremen, Germany; 2 DTU Nanotech, Department of Micro and Nanotechnology, Technical University of Denmark, Ørsteds Plads, Building 345E, DK-2800 Kongens Lyngby, Denmark

**Keywords:** fullerene, molecular spintronics, scanning tunneling microscopy, spin transport

## Abstract

Motivated by the recent achievements in the manipulation of C_60_ molecules in STM experiments, we study theoretically the structure and electronic properties of a C_60_ molecule in an STM tunneljunction with a magnetic tip and magnetic adatom on a Cu(111) surface using first-principles calculations. For the case of a vanadium tip/adatom, we demonstrate how spin coupling between the magnetic V atoms, mediated by the C_60_, can be observed in the electronic transport, which display a strong spin-filtering effect, allowing mainly majority-spin electrons to pass (>95%). Moreover, we find a significant change in the conductance between parallel and anti-parallel spin polarizations in the junction (86%) which suggests that STM experiments should be able to characterize the magnetism and spin coupling for these systems.

## Introduction

Organic materials typically offer small spin–orbit and hyperfine interactions, which are prerequisites for spintronic applications, because they allow long spin lifetimes. Thus there is a great interest in organic building blocks for molecular spintronics [1–4], and a thorough understanding of spin transport and magnetism in these systems is called for. It is therefore important to establish model molecular spintronic systems where spin transport and magnetic interactions can be examined experimentally. Recently, it has been demonstrated in low temperature scanning tunneling microscopy (STM) experiments how C_60_ molecules can be picked up by the STM-tip, and how they could controllably be used to contact structures such as adatoms, clusters, and molecules placed on a substrate surface [5–7]. The C_60_ molecule is considered as an attractive anchoring group for molecular electronics due to its mechanical robustness [8]. Moreover, the lowest unoccupied molecular orbital (LUMO) of C_60_ is close to the Fermi level of ferromagnetic elements which makes spin injection relatively easy [9], and characterizes C_60_ as a promising building block in molecular spintronics. The high symmetry of the C_60_ allows detailed characterization of the bonding geometries in STM. In particular, one can determine which part of C_60_ is pointing towards the tip/surface prior to contact formation, and also after contact formation, while the C_60_ is placed on the tip [7]. Subsequently, it is possible to investigate the interactions between tip and sample via electronic transport measurements as tip and sample are brought into contact. STM also provides a powerful tool for investigating spin-transport in magnetic nanostructures [10–17]. Direct magnetic interactions between STM tip and magnetic materials on a substrate have been studied in a number of works [18–20], and STM has been used to probe spin in organic molecules [21]. In the case of a magnetic tip and magnetic surfaces, this method may be used to study spin transport and interactions through organic molecular systems bound to the surface and gain insight into single-molecular magnetic properties. Among organic compounds, carbonic rings which are combined with transition metals are interesting for molecular spintronics purposes [22]. The interaction of the π-electron system of such rings with the d-orbitals of the transition metals, is a key to electron and subsequently to spin transport. One example of such systems is presented in a theoretical study, where calculations have been used to examine spin transport in a benzene–Co system on a Cu(001) surface contacted by a Cr tip [23]. The magnetic properties and spin transport have also been calculated for organometallic “multidecker” wires, where magnetic atoms are sandwiched between organic parts [24]. Multidecker systems involving vanadium are very promising due to their half-metallic behavior resulting in high spin polarization of the transport [24–26]. Interestingly, due to the different symmetries of the C_60_, it might be possible to vary the electronic and magnetic properties depending on whether pentagon, hexagon or edge sites of C_60_ are in contact with the magnetic ligand atoms.

Here, we employ first-principles calculations to predict the spin transport through a spintronic model system consisting of a C_60_ molecule contacted by magnetic atoms in an STM setup. In particular, we predict that vanadium is a magnetic material which will show pronounced spin-filtering and spin-valve effects in STM experiments.

## System setup and methods

**Figure 1:**
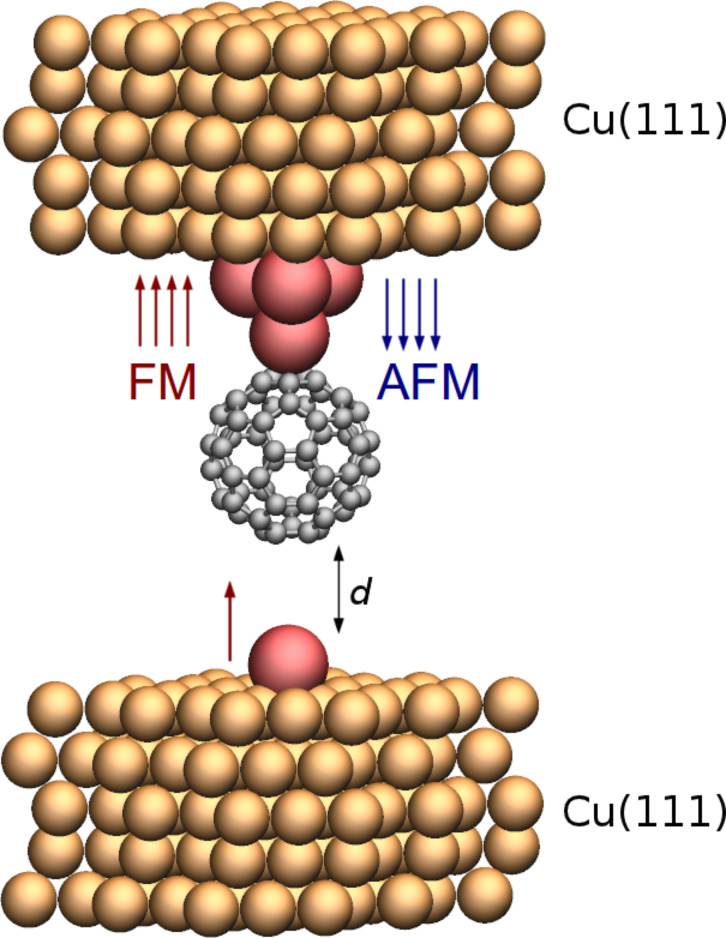
STM system picking up C_60_ with a magnetic tip, approaching a magnetic adatom on the nonmagnetic copper surface. The orange, red and gray spheres depict copper, vanadium and carbon atoms, respectively. A C_60_-pentagon is facing the tip/adatom.

In order to mimic a concrete STM experiment, we investigated the specific setup shown in Figure 1. The bulk regions of the contacts (i.e., STM tip and substrate) have been chosen to be nonmagnetic copper, which has previously been employed in manipulation experiments [5–7]. We imagine that magnetic atoms are deposited on the Cu surface prior to deposition [27] of the C_60_ molecules, and that the tip-electrode is prepared prior to the contact by either indenting a Cu tip into a cluster of these atoms in order to cover its outermost part with these, or by creating the tip from from the bulk magnetic material. We model the outermost part of the tip by a pyramid-like structure consisting of four atoms on one electrode, simulating the magnetic STM tip which is used to pick up C_60_ for subsequent contact formation to an isolated adatom on the copper(111) substrate. In the following we will show that the case of vanadium is remarkable. We used spin-polarized pseudopotential DFT calculations with the SIESTA code [28]. Electronic structures have been calculated within a GGA-PBE approximation to the exchange and correlation functional [29]. Double-ζ polarized basis sets with grid cutoff of 250 Ry have been used. Spin-polarized transport was subsequently calculated using the non-equilibrium Green’s function (NEGF) formalism [30] in the limit of zero voltage. In order to eliminate basis set superposition error (BSSE) present in methods with atomic orbitals basis sets, we have used plane-wave (PW) formalism as implemented in [31] for total energy calculations. In these calculations, we have been using ultrasoft pseudopotentials, with 30/300 Ry cutoff for wavefunction/charge density.

## Results and Discussion

As an initial rough guide in our search for interesting magnetic metals to contact the C_60_, and to select relevant bonding sites, we first performed calculations of the binding energy of a single adatom from the first row transition metals (from vanadium to copper) with C_60_. This will clearly overestimate the binding of the C_60_ to a higher coordinated tip-atom but we are here focussing on the trends in binding energy depending on tip-atom species. Based on the simple adatom calculations we seek high magnetization and a high binding energy to get a stable contact. The results are summarized in Table 1 for different sites on the C_60_ molecule. It can be seen that nickel has the strongest binding energy but with zero total magnetization (*M*_T_), and thus, is probably not interesting for investigations of spin transport. On the other hand, chromium enjoys the largest *M*_T_, due to its largest unpaired electronic configuration [Ar]3d^5^4s^1^ but with the least binding energy strength. It has already been shown that copper STM tips can pick up C_60_ [5]. Noting that the maximum of binding energy for copper to C_60_ is ≈0.8 eV, we conclude that the same action should be possible with vanadium while enjoying a decent *M*_T_.

**Table 1:** Binding energy 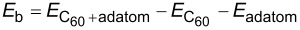 and total magnetization *M*_T_ = ∫*_v_*(*n*_up_ − *n*_down_)*dr*^3^ per unit cell. The distance between the adatom and C_60_ has been optimized.

adatom	56*^a^* (eV)	66*^b^* (eV)	hexagon (eV)	pentagon (eV)	top (eV)	*M*_T_

V	−1.13	−1.25	−0.93	−1.40	−1.06	5.00
Cr	−0.58	−0.46	1.77	2.74	−0.49	6.00
Mn	−0.39	−0.58	−0.25	−0.33	−0.40	5.00
Fe	−0.83	0.08	−0.87	−0.55	−0.75	4.00
Co	−0.90	−1.22	−0.87	−0.88	−0.80	1.00
Ni	−1.48	−1.64	−1.24	−1.44	−1.26	0.00
Cu	−0.79	−0.60	−0.08	−0.34	−0.74	0.00

*^a^*56 refers to sites above edges shared between a hexagon and a pentagon and top means top of a carbon atom. *^b^*66 refers to sites above edges shared between two hexagons.

**Figure 2:**
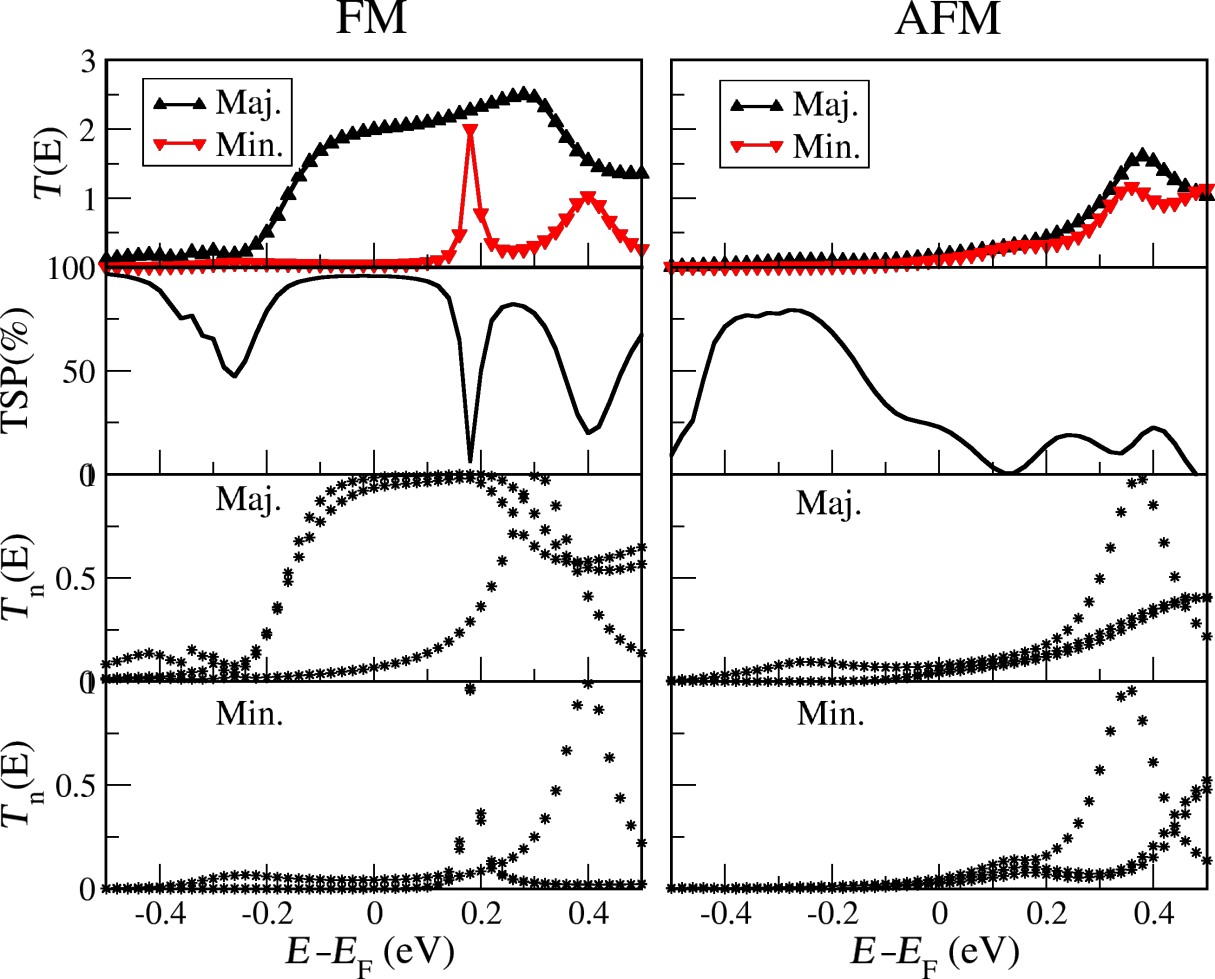
Transmission spectra for FM and AFM arrangements. The first row shows spin-resolved transmission spectra for each arrangement. The second row demonstrates the corresponding transmission spin polarization as defined in the text. The third and forth rows show transmission eigenvalues for three channels in each spin component.

Based on the data of the simple guiding calculations we chose vanadium in the full C_60_-contact simulations on the system depicted in Figure 1. In the following we demonstrate that this choice for contact material in an STM setup is successful in achieving a good spin-filter- and spin-valve performance. Here, ferromagnetic (FM) and anti-ferromagnetic (AFM) spin alignment between atoms of tip and adatom, have been considered. The site on the C_60_ with the highest binding energy for a V adatom is η^5^, which is roughly over the center of a pentagon of a C_60_, and due to the symmetric structure of the C_60_, this site is contacted by both the tip and the adatom. We find that the binding energy of the C_60_ to the V-tip (upper part in Figure 1) is 1.3 eV, while binding of C_60_ to the adatom on the Cu substrate (lower part in Figure 1) is 1.1 eV. The spin-resolved transmissions for the FM and AFM cases are shown in Figure 2. We first focus on the highly conducting contact configuration where the atomic structure of the C_60_ along with vanadium atoms and first copper layers of both sides have been relaxed to the force threshold of 0.05 eV/Å. We also show the transmission spin polarization (TSP), defined as

[1]
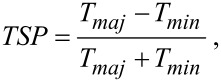


and channel decomposed transmission values in Figure 2. Here, we first point out a remarkable spin-filtering effect in the FM arrangement, whereby two almost open channels conduct in the vicinity of the Fermi level for the majority spin component, while the minority channels are almost closed. For the minority spin component the resonance peaks at ≈0.2 and 0.4 eV produce dips in the corresponding TSP curve, however, these will only be of importance for a voltage bias comparable to these energies. Transmission eigenvalues of the first three dominant channels are shown in the third and forth panels, that clearly show two distinct channels for the FM-majority spin channels. Furthermore, it is striking that the channels in the AFM configuration are almost closed, except for small resonance peaks at ≈0.35 eV, which again only will come into play for higher voltages.

**Figure 3:**
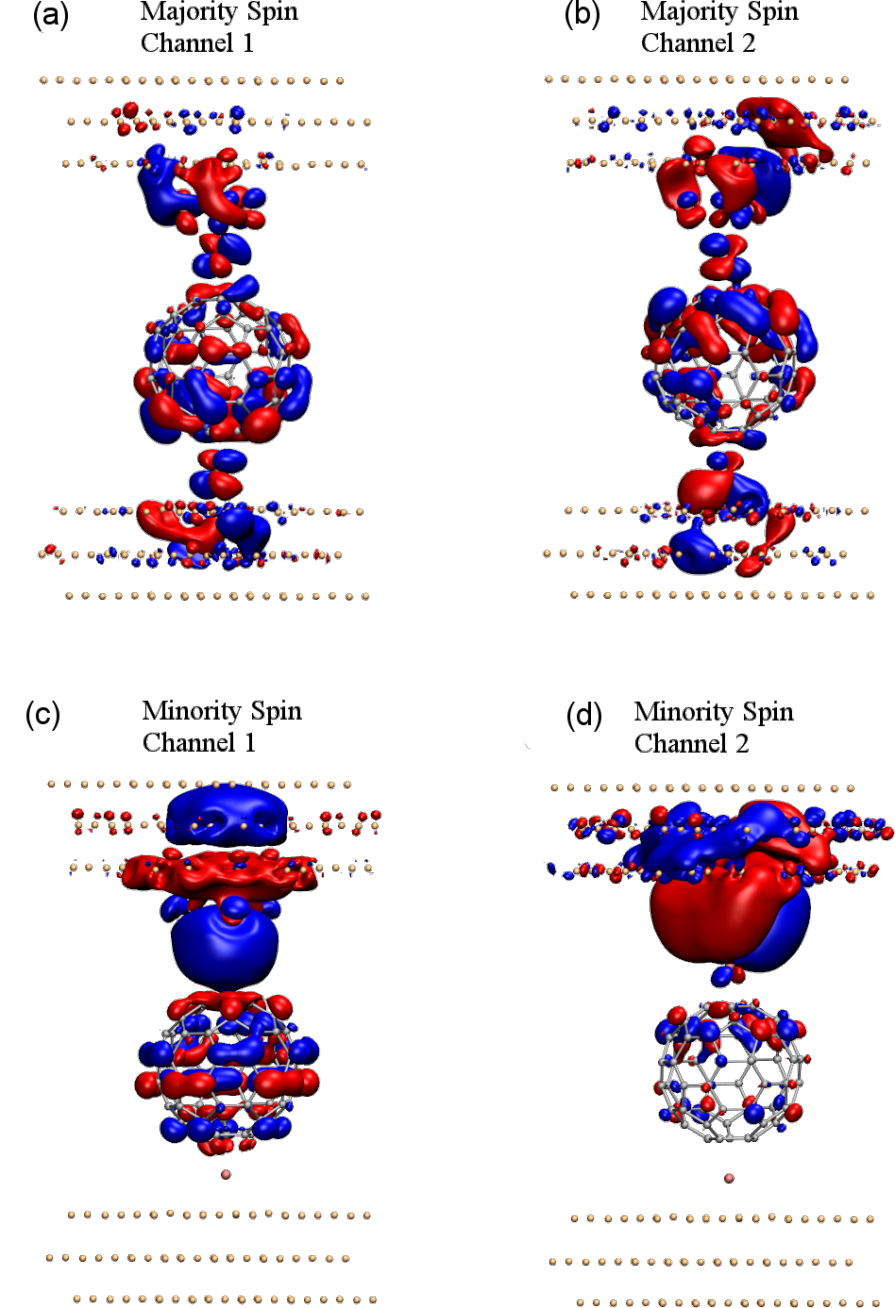
Scattering states at *E* = *E*_F_ of first two dominant eigenchannels for (a,b) majority and (c,d) minority spin components in FM arrangement. Blue and red indicate the positive and negative sign of the real part of the wavefunction.

To better understand the nature of spin transport in the system, we have calculated the spatially-resolved scattering states in the contact region [32]. The results are shown in Figure 3. Here, we consider the conducting FM arrangement and focus on the two eigenchannel scattering states with highest transmission at *E*_F_ (moving in the direction up-to-down), which both are almost fully transmitting. For the majority spins, we notice the *d_zx_* and *d_yz_* orbital nature of wavefunctions on the V adatoms contacting C_60_ (*z* chosen perpendicular to the surface). This is in accordance with the Mulliken population analysis of the majority spin states of the V tip and adatoms, where the *d_zx_* and *d_yz_* each appears half-filled. On the other hand, the *d_xy_*, 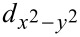 and 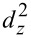 are closer to being filled, while the *s* is closer to being empty. This points to a charge transfer from the V atoms to the C_60_ leaving the *d_zx_*/*d_yz_* orbital energies closest to *E*_F_. Since the *d_zx_*/*d_yz_* orbitals match the symmetry with angular momentum *m* = 1 for rotation around the V–C_60_–V axis of the pentagon-prone 3-fold degenerate LUMO states (t_1_*_u_* symmetry [33–34]), we can expect the observed orbitals in the transport channels. For minority spins *d_zx_* and *d_yz_* orbitals are almost empty and shifted away from *E*_F_, resulting in a vanishing transmission. The rotational symmetric *m* = 0 channels appear as resonances in the channel transmissions above *E*_F_ and thus play a minor role.

**Figure 4:**
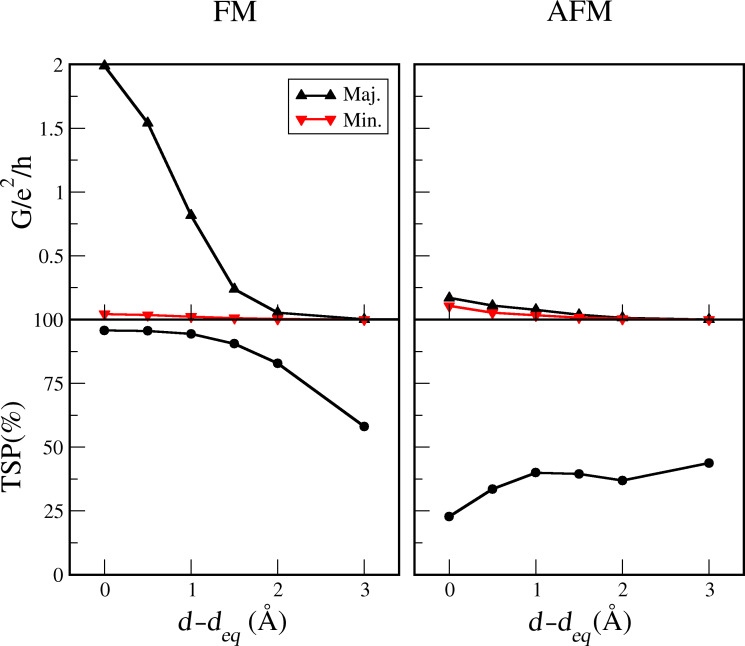
Spin-resolved conductance and transmission spin polarization (TSP) vs C_60_-adatom separation.

In typical STM experiments the conductance is probed from the tunnel-regime to contact. We have performed transport calculations as the tip is approaching the surface adatom until the tip–molecule distance (*d* shown in Figure 1) approximately reaches the equilibrium distance discussed above. In Figure 4 we display the conductance along with the corresponding TSP as a function of the tip distance. As can be seen, there is a trend of an increasing conductance of the majority spins and thus TSP, in the FM case, while in the AFM case, the conductance values are considerably smaller all the way to the equilibrium contact distance.

**Figure 5:**
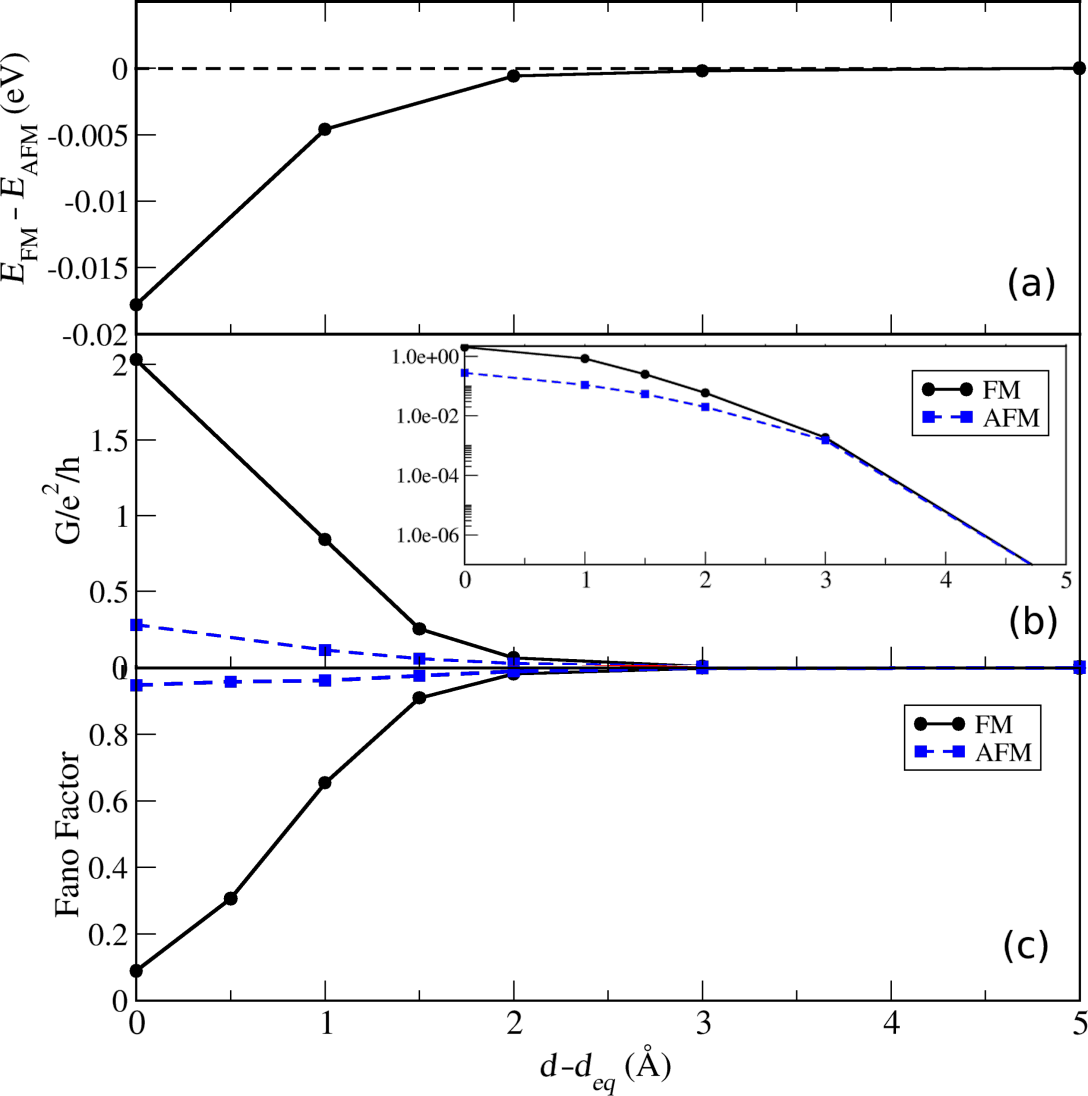
(a) Magnetic exchange energy, (b) conductance for FM and AFM configurations (inset in log-scale) and (c) Fano factor of transmission as a function of C_60_-adatom separation for the FM and AFM configurations.

The difference between the FM and AFM conductance properties indicates how it is possible to probe the spin coupling mediated by the C_60_ between the magnetic tip and substrate. The calculated magnetic interaction between the tip and adatom, the magnetic exchange energy defined as *E*_FM_ − *E*_AFM_, is shown in Figure 5a when the tip molecule is approaching the adatom. This shows that the FM arrangement becomes favorable as the molecule reaches the equilibrium distance to the surface adatom. To be sure about the fidelity of the values obtained here, we have performed the same study using the PW method. We found that the trend is the same and that the values are even more pronounced in favor of FM arrangement, though of the same order of magnitude.

A graph of the total conductance versus C_60_-adatom distance is shown in Figure 5b. The conductance behavior demonstrates a *magnetic* valve, being closed for FM and open for AFM, if we imagine an external control over the magnetization of tip/substrate. In a typical experiment with a bulk magnetic tip the magnetization of the tip will be determined by the intrinsic magnetic anisotropy of the crystalline magnetization, which fixes the magnetization axes. As the tip molecule approaches the adatom on the non-magnetic surface, its magnetization will be determined by the interaction with the tip mediated by the molecule. In this case the adatom magnetization will align according to the thermal occupations.

The absolute distance is typically not known in an actual STM experiment. In principle, a particular conductance could be realized with both FM or AFM spin configurations – a conductance of *e*^2^/*h* could result from a single spin-channel with perfect transmission or two half-transmitting channels. In combination with measurements of the conductance, measurements of current shot-noise as characterized by the Fano factor,

[2]
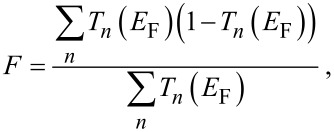


can provide further insights into the distribution of transmissions in the conductance channels as demonstrated for molecular contacts [35–36]. In Figure 5c, we observe how the noise is significantly smaller for the FM configuration and drops already well before contact (*d* − *d_eq_* ≈ 1.5 Å) is established. Since the shot noise in the FM case is low in contact, while the conductance is close to 2*G*_0_, it can be inferred from this that the transport is carried by two almost perfectly transmitting channels in the FM contact configuration.

## Conclusion

We have performed first-principles spin-polarized density functional calculations and investigated the electron transport properties of a C_60_-molecular junction in a setup relevant for STM experiments. Our results demonstrate how the FM and AFM configurations can be identified due to their markedly different conductance and shot noise. Thus, it may allow for the study of the magnetic coupling between tip and substrate mediated by the molecule as the contact is being formed. For the FM case only the majority channels contribute to transport and the system act as a spin-filter. This is similar to what has been predicted for vanadium multidecker systems [25–26], but the STM setup we propose here might be more accessible for experiments.

## References

[1] Xiong Z H, Wu D, Vardeny Z V, Shi J (2004). Nature.

[2] Rocha A R, García-Suárez V, Bailey S W, Lambert C J, Ferrer J, Sanvito S (2005). Nat Mater.

[3] Koopmans B, Wagemans W, Bloom F L, Bobbert P A, Kemerink M, Wohlgenannt M (2011). Philos Trans R Soc, A.

[4] Herrmann C, Solomon G C, Ratner M A (2010). J Am Chem Soc.

[5] Schull G, Frederiksen T, Brandbyge M, Berndt R (2009). Phys Rev Lett.

[6] Berndt R, Kröger J, Néel N, Schull G (2010). Phys Chem Chem Phys.

[7] Schull G, Frederiksen T, Arnau A, Sánchez-Portal D, Berndt R (2011). Nat Nanotechnol.

[8] Martin C A, Ding D, Sørensen J K, Bjørnholm T, van Ruitenbeek J M, van der Zant H S J (2008). J Am Chem Soc.

[9] Braun S, Salaneck W R, Fahlman M (2009). Adv Mater.

[10] Bode M (2003). Rep Prog Phys.

[11] Wiesendanger R, Güntherodt H-J, Güntherodt G, Gambino R J, Ruf R (1990). Phys Rev Lett.

[12] Hirjibehedin C F, Lutz C P, Heinrich A J (2006). Science.

[13] Néel N, Kröger J, Berndt R (2010). Phys Rev B.

[14] Liljeroth P, Swart I, Paavilainen S, Repp J, Meyer G (2010). Nano Lett.

[15] Iacovita C, Rastei M V, Heinrich B W, Brumme T, Kortus J, Limot L, Bucher J P (2008). Phys Rev Lett.

[16] Brede J, Atodiresei N, Kuck S, Lazić P, Caciuc V, Morikawa Y, Hoffmann G, Blügel S, Wiesendanger R (2010). Phys Rev Lett.

[17] Schmaus S, Bagrets A, Nahas Y, Yamada T K, Bork A, Bowen M, Beaurepaire E, Evers F, Wulfhekel W (2011). Nat Nanotechnol.

[18] Tao K, Stepanyuk V S, Hergert W, Rungger I, Sanvito S, Bruno P (2009). Phys Rev Lett.

[19] Polok M, Fedorov D V, Bagrets A, Zahn P, Mertig I (2011). Phys Rev B.

[20] Brune H, Gambardella P (2009). Surf Sci.

[21] Atodiresei N, Brede J, Lazić P, Caciuc V, Hoffmann G, Wiesendanger R, Blügel S (2010). Phys Rev Lett.

[22] Crabtree R (2009). The organometallic chemistry of the transition metals.

[23] Tao K, Stepanyuk V S, Bruno P, Bazhanov D I, Maslyuk V V, Brandbyge M, Mertig I (2008). Phys Rev B.

[24] Wang L, Cai Z, Wang J, Lu J, Luo G, Lai L, Zhou J, Qin R, Gao Z, Yu D (2008). Nano Lett.

[25] Maslyuk V V, Bagrets A, Meded V, Arnold A, Evers F, Brandbyge M, Bredow T, Mertig I (2006). Phys Rev Lett.

[26] Koleini M, Paulsson M, Brandbyge M (2007). Phys Rev Lett.

[27] Weber S E, Rao B K, Jena P, Stepanyuk V S, Hergert W, Wildberger K, Zeller R, Dederichs P H (1997). J Phys: Condens Matter.

[28] Soler J M, Artacho E, Gale J D, García A, Junquera J, Ordejón P, Sánchez-Portal D (2002). J Phys: Condens Matter.

[29] Perdew J P, Burke K, Ernzerhof M (1996). Phys Rev Lett.

[30] Brandbyge M, Mozos J-L, Ordejon P, Taylor J, Stokbro K (2002). Phys Rev B.

[31] Giannozzi P, Baroni S, Bonini N, Calandra M, Car R, Cavazzoni C, Ceresoli D, Chiarotti G L, Cococcioni M, Dabo I (2009). J Phys: Condens Matter.

[32] Paulsson M, Brandbyge M (2007). Phys Rev B.

[33] Haddon R C, Brus L E, Raghavachari K (1986). Chem Phys Lett.

[34] Hands I D, Dunn J L, Bates C A (2010). Phys Rev B.

[35] Djukic D, van Ruitenbeek J M (2006). Nano Lett.

[36] Kiguchi M, Tal O, Wohlthat S, Pauly F, Krieger M, Djukic D, Cuevas J C, van Ruitenbeek J M (2008). Phys Rev Lett.

